# Recent advances in the management of priapism

**DOI:** 10.12688/f1000research.12828.1

**Published:** 2018-01-10

**Authors:** Asif Muneer, Hussain M. Alnajjar, David Ralph

**Affiliations:** 1 Department of Urology, University College London Hospitals NHS Trust, London, UK; 2 NIHR Biomedical Research Centre, University College London Hospitals NHS Trust, London, UK

**Keywords:** Priapism, Penile dysfunction, Urological

## Abstract

Priapism is an uncommon urological emergency that can lead to permanent impotence if prompt presentation and medical intervention is not performed. It is a breakdown of the usual physiological mechanisms controlling penile tumescence and detumescence, leading to a prolonged penile erection (>4 hours) that is unrelated to sexual stimulation. Currently, there are three accepted subtypes: ischaemic, non-ischaemic, and stuttering priapism, which is also known as recurrent ischemic priapism. The aim of treatment is the immediate resolution of the painful erection and the preservation of cavernosal smooth muscle function in order to prevent cavernosal fibrosis, which can lead to penile shortening and permanent erectile dysfunction.

## Introduction

Priapism is a rare urological emergency that can result in long-term erectile dysfunction if prompt presentation and medical intervention is not performed. Priapism is a disruption related to the usual physiological mechanisms controlling penile tumescence and detumescence, leading to a prolonged penile erection that is unrelated to sexual stimulation ^1^.

Currently, there are three accepted subtypes: ischemic or low-flow priapism, non-ischemic or high-flow priapism, and stuttering priapism, also known as recurrent ischemic priapism ^1– 4^. For the purposes of consistency, the terms ischaemic and non-ischaemic are preferred over low flow and high flow, particularly when interpreting penile imaging following therapeutic interventions and it also ensures that clinicians appreciate the urgency of intervention.

The aim of treatment is the immediate resolution of the painful erection and the preservation of cavernosal smooth muscle function in order to prevent long-term penile shortening and refractory erectile dysfunction secondary to cavernosal fibrosis.

There has been very little in the way of new developments in priapism, with the majority of novel concepts related to early implantation of a penile prosthesis and a better understanding of the imaging modalities. Some of these advances will be discussed below.

## Advances in priapism diagnostics

The clinical history and examination can be supplemented by radiological imaging and blood gas analyses from the corpus cavernosum, which in most cases will provide a diagnosis and differentiation between the different subtypes. Further investigations using urine toxicology, haematological screening, and abdominal imaging is performed to investigate an underlying cause, although the majority of ischaemic priapism cases are likely to be idiopathic ( Table 1).

**Table 1.** Causes of priapism.

Priapism type	Causes
Ischaemic priapism	Idiopathic, haematological dyscrasias (e.g. sickle cell anaemia, thalassemia, and leukaemia), illicit drugs (e.g. cocaine and marijuana), medications (e.g. antipsychotics, antidepressants, intracavernosal prostaglandin E1 injections, anticoagulants, and alpha blockers), pelvic malignancy, neurological disorders (e.g. spinal cord injury), toxic infections (e.g. scorpion sting), and metabolic disorders (e.g. gout, amyloidosis).
Non-ischaemic priapism	Trauma to penis/perineum, treatment of ischaemic priapism *.

*The other mechanism for non-ischemic priapism is in the management of low-flow priapism that requires injections. It is often reported that the needles themselves can transect or damage cavernosal arteries, thus creating a fistula *de novo* and converting what was an ischaemic priapism into a non-ischaemic scenario.

### Clinical history

Ischaemic priapism often presents well beyond the four-hour time frame commonly used as the time interval in international guidelines whereby smooth muscle necrosis within the corpus cavernosum commences ^5^.

Non-ischaemic priapism can have a preceding history of blunt or penetrating perineal or genital trauma, which may have occurred several weeks before the onset of priapism. Penile discomfort rather than severe penile pain is often reported by patients; however, accurately distinguishing this condition from ischaemic priapism, which requires prompt intervention, is imperative.

### Radiological imaging in priapism

Assessment of blood flow within cavernosal arteries and the corpus cavernosum to distinguish ischaemic from non-ischaemic priapism subtypes can be performed using colour penile Doppler ultrasonography. Impaired blood flow of the distal corpus cavernosum and reduced or absent perfusion within the cavernosal arteries is seen in ischaemic priapism cases on colour penile Doppler imaging. However, if blood has already been aspirated from the corpora, interpreting penile Doppler studies can be problematic because of the development of abnormal aberrant blood flow in sections of the corpus cavernosum ^6^. In non-ischaemic priapism, the Doppler studies will demonstrate a high velocity throughout the corpus cavernosum and often the demonstration of a fistula. A recent study interpreting the Doppler waveforms shows that the interpretation is often difficult once interventions have been undertaken ^7^.

### Blood gas analysis

A more reliable investigation to distinguish between ischaemic and non-ischaemic priapism is to aspirate blood from the corpus cavernosum for blood gas analysis prior to any other intervention ^8^. This will confirm blood with a low pO _2_ and acidosis if the diagnosis is ischaemic priapism or a normal pO _2_ and normal pH in cases of non-ischaemic priapism ( Table 2).

**Table 2.** Blood gas findings for ischaemic and non-ischaemic priapism.

	pO _2_ mmHg	pCO _2_ mmHg	pH
Ischaemic priapism	<30	>60	<7.25
Non-ischaemic priapism	>90	<40	7.35

## Management of ischaemic priapism

If the duration of priapism is between 4 and 24 hours, the initial conservative management involves pain management, encouraging ejaculation, vigorous physical exercise, and ice packs with the aim to induce smooth muscle contraction via sympathomimetic α receptors and thus induce detumescence. Failing this, the following steps should be used to manage ischaemic priapism:

### i) Aspiration and instillation of α agonists into the corpus cavernosum

Following a penile block, if aspiration of ischaemic blood fails to resolve the priapism, instillation of α-adrenergic agonists (usually 200 μg of phenylephrine repeated to a maximum of 1,500 μg) is the next step. Both aspiration and injection of α-agonists is performed via a large bore needle (19G) inserted through the glans penis and into the corpora or directly into the penile shaft at the 2 or 10 o’clock positions to avoid injuring the neurovascular bundles. This manoeuvre aims to increase the smooth muscle tone of the corpora cavernosa and promote detumescence.

Alternative α-adrenergic agonists include metaraminol and adrenaline. High-dose phenylephrine has also been successfully utilised in small case series ^9^, although in refractory cases it is unlikely to be successful owing to the development of irreversible smooth muscle dysfunction ^5, 10^. Ischaemic blood aspiration alone may remedy the ischaemic priapism in up to-one third of cases; therefore, it should always be the first approach before administering phenylephrine because of the impairment of smooth muscle contraction by the development of an ischaemic microenvironment ^11, 12^. Phenylephrine injection should be performed with continuous monitoring of blood pressure, especially in patients with pre-existing hypertension, as it may potentially precipitate a cardiovascular event.

Priapism episodes lasting more than 24 to 36 hours are less likely to respond to corporal blood aspiration and instillation of α-adrenergic agonists because of the presence of irreversible smooth muscle damage. However, it is essential to perform the above manoeuvres in all patients presenting with ischaemic priapism irrespective of the time of presentation. This is because the absolute time point at which permanent smooth muscle damage occurs is not clear, although hypoxia, glucopenia, and acidosis usually develop within six hours of the commencement of ischaemic priapism.

If ischaemic priapism is reversed within 24 hours, there is usually a recovery of erectile function in approximately 50% of patients, while a duration of more than 36 hours of the priapism episode is invariably associated with a degree of corporal fibrosis and erectile dysfunction ^13^. The outcomes in the time period of 24 to 36 hours are variable and governed by the degree of ischaemia and the amount of reversible smooth muscle function if penile detumescence is achieved.

Both *in vitro* and *in vivo* studies have shown that hypoxia and glucopenia may function as independent factors causing cavernosal smooth muscle dysfunction and that irreversible histopathological alterations are caused by the combination of acidosis, glucopenia, and hypoxia that last for more than four hours ^5^.

Histologically, as priapism continues, the corpus cavernosum undergoes progressive changes. In cases where priapism is of a short duration (less than 12 hours), the tissue consists of minor endothelial defects with occasional lymphocytic infiltration but the smooth muscle cells remain intact. It is only after 12 to 14 hours of ischaemic priapism that the trabecular smooth muscle cells show the beginning of focal cytoplasmic transformation, with the perinuclear cytoplasm, endoplasmic reticulum, ribosomes, and Golgi apparatus exhibiting an increase in size. In the time period of 24 to 48 hours, there is widespread endothelial destruction and exposure of the basement membrane. In addition to this, the smooth muscle cells undergo transformation as well as necrosis. Persistent blood stasis for over two days is related to the infiltration of the trabecular tissue with inflammatory cells and the smooth muscle cells undergoing necrosis or phenotypically changing into fibroblast-like cells ^14^.

At the molecular level, decreases in phosphodiesterase type 5 (PDE-5) and RhoA/Rho kinase, increased levels of adenosine, and the presence of acidosis, glucopenia, and hypoxia are all likely to contribute to cavernosal smooth muscle dysfunction ^15^.

### ii) Management of priapism using surgical shunts

For those patients in whom corporal blood aspiration and instillation of α-adrenergic agonists are ineffective, surgical shunts are frequently required. The concept of surgically created shunts is to allow the drainage of ischaemic blood from the corpora cavernosa to either the corpus spongiosum or the saphenous vein, although the older shunts (proximal) described are no longer routinely used.

Minimally invasive distal percutaneous shunts such as The Winter and Ebbehoj can be used. The Winter shunt involves the insertion of a large bore needle or a cannula into the glans penis and the distal end of the corpus cavernosum. The Ebbehoj procedure entails a stab incision with a No. 10 scalpel blade into the corpora cavernosa through the glans penis. When percutaneous shunt surgery is unsuccessful, an Al-Ghorab shunt, which is an open corporoglanular shunt involving the excision of a segment of tunica albuginea from the tip of the corpus cavernosum, has been described.

The most recent shunt techniques aim to allow better communication between the distal corpora and glans penis. Described as a T-shunt, the technique involves the insertion of a No. 10 blade through the glans penis into the ipsilateral corpus cavernosum followed by a 90-degree rotation laterally away from the urethra to create a fistula ^16^. If detumescence is not achieved, the procedure can be redone on the contralateral side (TT shunt technique). In cases where TT shunt has failed, a tunnelling manoeuvre may be attempted; this technique allows the ischaemic blood to be drained from the proximal aspect of the corpora cavernosa. This procedure, also known as the corporal snake manoeuvre, uses a 22 French urethral sound through the previous T-shunt ^17, 18^.

Although early reports demonstrate the safety of a distal shunt coupled with the tunnelling manoeuvre, with resolution achieved in nearly all cases ^17^, a recent series of 45 patients has shown that the success of the T shunt and tunnelling manoeuvre is dependent on the duration of the priapism ^19^.

### iii) Implantation of a penile prosthesis in acute priapism

If aspiration of ischaemic blood from the corpora cavernosa followed by repeated instillations of α-adrenergic agonists does not induce resolution of the priapism episode, irreversible damage to the smooth muscle is likely to have already occurred. Consequently, efforts to re-oxygenate the corpora with shunt surgery, even though it could resolve the painful priapism episode, will not prevent the onset of refractory erectile dysfunction in the long term, which will ultimately require the implantation of a penile prosthesis ^20^.

Penile prosthesis implantation, which has been the traditional treatment in patients who have developed erectile dysfunction following a prolonged priapism, has now been advocated as an alternative initial management strategy for prolonged ischaemic priapism ^21, 22^.

In fact, urgent penile prosthesis implantation in cases of refractory priapism and cavernosal smooth muscle necrosis reduces the painful priapism occurrence, assures the necessary long-term rigidity required for sexual intercourse, and stops penile shortening and curvature secondary to the development of corporal fibrosis, which would otherwise be unavoidable.

Potential overtreatment of patients with no evidence of necrosis in the cavernosal smooth muscle is one of the risks associated with this approach, and therefore the correct timing of surgery is paramount. Although after 24 to 48 hours of a persistent erection irreversible smooth muscle necrosis has already developed, the use of additional imaging modalities and cavernosal smooth muscle biopsies aid clinicians in the decision of whether or not an early penile prosthesis is suitable or whether a more conservative approach can be used with the hope that there is cavernosal smooth muscle recovery in the long term. In a series of 23 patients, in which the radiological findings have been correlated with biopsies from the corpus cavernosum, gadolinium-enhanced high-definition magnetic resonance imaging (MRI) of the penis demonstrated a sensitivity of 100% when utilised in the detection of necrosis of the cavernosal smooth muscle ^23^. Therefore, this imaging modality may be extremely useful in supporting the clinical decision to proceed with acute penile prosthesis implantation rather than adopt a conservative policy. Therefore, MRI imaging with diagnostic biopsies aid decision making in these difficult cases.

Implantation of a malleable prosthesis is easier in the early period (first two weeks) of refractory ischaemic priapism, as corporal fibrosis has not been completely established. At a later date, the malleable prosthesis can be exchanged for an inflatable prosthesis, this also allows upsizing of the cylinders ^24^.

In challenging cases where the corpora are severely fibrosed, simultaneous penile prosthesis implantation and corporal reconstruction has been shown to offer satisfactory results. However, patients must be fully counselled and made aware that complication rates are significantly higher in these complex cases because of the severe fibrosis ^25^. Several other techniques have been described (e.g. multiple corporal incisions, corporal excavation, and downsized prosthesis) in order to minimize complications and improve surgical outcomes ^26^.

## Update on the management of ischaemic and non-ischaemic priapism

Daily low-dose PDE-5 inhibitor treatment in a small series of patients prevented recurrent priapism while preserving normal erectile function. Burnett *et al*. reported this interesting therapy to prevent recurrent ischaemic priapism (stuttering) in a study providing evidence to support the role of nitric oxide synthase/PDE-5 dysregulation as an essential factor in the pathogenesis of priapism ^27^.

The presence of oxygenated blood within the corpora and the lack of severe penile pain allows non-ischaemic cases to be managed conservatively provided that the initial diagnosis is accurate. After a conservative treatment approach, which requires regular clinical review, has been employed, diagnostic angiography combined with super-selective embolization of any fistula can be performed if the fistula has not yet closed spontaneously. Occasionally, patients can develop fibrosis within the distal corpora in long-standing non-ischaemic priapism and therefore early intervention with embolization is advocated to prevent this. Fibrosis within the distal corpus cavernosum can present as distal flaccidity and is best imaged using a penile MRI scan ^28^.
